# Evaluation of the delivered activity of yttrium-90 resin microspheres using sterile water and 5 % glucose during administration

**DOI:** 10.1186/s13550-015-0133-z

**Published:** 2015-10-13

**Authors:** Hojjat Ahmadzadehfar, Carsten Meyer, Claus Christian Pieper, Ralph Bundschuh, Marianne Muckle, Florian Gärtner, Hans Heinz Schild, Markus Essler

**Affiliations:** Department of Nuclear Medicine, University Hospital Bonn, Sigmund-Freud-Str. 25, 53127 Bonn, Germany; Department of Radiology, University Hospital Bonn, Bonn, Germany

**Keywords:** Radioembolization, SIR-Spheres, Resin microspheres, Glucose 5 %, Stasis, Backflow, Yttrium-90

## Abstract

**Background:**

The purpose of this study is to evaluate the impact of switching from sterile water to 5 % glucose (G5W) for the administration of yttrium-90 (^90^Y)-resin microspheres on the total activity of ^90^Y administered (expressed as a proportion of the prescribed/calculated activity), as well as the number of cases of stasis and the reported incidence of discomfort during the selective internal radiation therapy (SIRT) procedure.

**Methods:**

In December 2013, we switched from sterile water to G5W for the administration of SIRT using ^90^Y resin microspheres in all patients. This retrospective observational single-center case series describes our experience in the months preceding and after the switch. Apart from the change in administration medium, the protocol for SIRT was otherwise identical.

**Results:**

One hundred and four SIRT procedures were performed on 78 patients (45 male, mean age: 63 years, range: 31–87 years) with either unresectable hepatocellular carcinoma, cholangiocarcinoma, or chemorefractory liver-dominant metastatic cancer. Compared with sterile water, the whole prescribed activity was administered in significantly more procedures with G5W: 85 vs. 22 %; *p* < 0.0001. A significantly higher proportion of the calculated activity was administered with G5W: 96.1 ± 11.0 % vs. 77.4 ± 24.3 % (*p* < 0.0001). G5W procedures were also associated with a significantly lower incidence of stasis (28 vs. 11 % procedures; *p* = 0.02) and mild-to-moderate upper abdominal pain during the procedure (1.8 vs. 44 % procedures; *p* < 0.0001).

**Conclusions:**

Replacing sterile water with isotonic G5W during administration favorably impacts on the safety of SIRT, eliminates and/or minimizes flow reductions and stasis/reflux during administration of ^90^Y resin microspheres, improves percentage activity delivered, and reduces peri-procedural pain.

## Background

Selective internal radiation therapy (SIRT) is a form of liver-directed brachytherapy whereby microspheres loaded with yttrium-90 (^90^Y) serve as sealed sources of localized beta radiation [1]. The microspheres are delivered via the tumor-feeding arteries and embed permanently in the pre-capillary arterioles of liver tumors [2]. The goal of SIRT is to deliver potent beta radiation only to tumor cells via the enhanced arterial blood supply of the tumor so that there is minimal damage to the surrounding (healthy) liver parenchyma and negligible microsphere migration to other organs [1, 3]. One vial of 3 GBq ^90^Y resin microspheres contains between 40 and 80 million micron-sized microspheres [4]. The resin microspheres have a specific gravity close to plasma and so are neutrally buoyant [5] and minimally embolic (compared with large particles such as those used for transarterial chemoembolization (TACE)) [6]. The resin microspheres, as a result of their specific gravity, rely on sufficient blood flow distal to the catheter tip for their delivery and distribution to the tumor sites. Equally, the localized radiation effect [7] is enhanced because the oxygen supply to the tumor is maintained, and so, the primary mechanism of SIRT is internal radiation rather than vascular embolization causing ischemia [8]. The physical features of the microspheres are also important to reduce the risk for stasis of blood flow [9] and the risk of potential microsphere reflux into the normal parenchyma or to the gastrointestinal tract.

Computer modeling of flow dynamics after microsphere release shows that targeted delivery (whether whole liver, lobar, segmental, or sub-segmental) is determined by the specific positioning of the microcatheter and blood flow in the hepatic arterial tree [5, 10]. In the clinic, catheter-directed therapies such as SIRT are evaluated using real-time image guidance using digital subtraction angiography (DSA) together with an injectable liquid non-ionic contrast media (CM) which enable the interventional radiologist to ensure the correct catheter position and assessment of vessel behavior, such as spasm or dissection. The infusion of ^90^Y resin microspheres under direct fluoroscopic guidance also enables the visualization of blood flow direction and velocity around the catheter and so ensures their safe delivery.

A standardized approach for the delivery of ^90^Y resin microspheres has evolved worldwide over time with periodic publication of updated guidance [4, 11]. This guidance is based on a process of regular review and/or formalized audit to ensure the optimal outcomes with this technique. One observed effect of SIRT in some patients is early stasis in which *the delivery of the whole calculated activity of resin microspheres was halted as a result of a lack of antegrade flow at angiography during infusion* which is sporadically reported in the literature [12]. Although stasis can impede delivery of the full prescribed activity of ^90^Y, two separate published studies have found that stasis neither affected early response outcomes as assessed by computed tomographic (CT) at 3 months post-SIRT [12] nor were there differences in survival when actual dose administered was either above or below a target of 80 % of the prescribe dose [13]. Nevertheless, mechanisms to improve the delivery of SIRT and reduce the potential for stasis continue to evolve.

The administration of SIRT using ^90^Y resin microspheres is normally carried out with sterile water. It has been hypothesized that the flush of sterile water may cause a temporary change in the osmolality of the blood leading to hemolysis [14] and vascular endothelial injury with subsequent vasospasm and premature stasis [15–17]. In some leading clinics, glucose 5 % water (G5W) in combination with non-ionic contrast medium is currently used instead of sterile water for the administration of ^90^Y resin microspheres because of its approximate isotonicity to plasma. Although there is anecdotal data from these centers to suggest that G5W may improve the efficacy and safety of SIRT by reducing the incidence of stasis and improving the activity of ^90^Y administered, the experience of these clinics using G5W is yet to be published.

The decision to suspend ^90^Y resin microspheres in G5W was based on unpublished data from Sirtex Medical Ltd. which examined the compatibility of SIR-Spheres® microspheres with non-ionic contrast media and fluids suitable for intravascular use other than sterile water. These in vitro studies found that G5W (isotonic) had equivalent compatibility to sterile water (hypotonic) with similar binding affinity of ^90^Y to the resin substrate.

In December 2013, we switched from sterile water to G5W for application of resin microspheres in all patients. This paper describes our experience with SIRT (in the months preceding and after the change to G5W) with respect to the total activity administered (expressed as a proportion of the calculated activity) and the number of cases of stasis and flow reduction as well as the reported incidence of discomfort during the application of SIRT.

## Methods

### Patients

This was a retrospective observational single-center case series evaluation of 78 patients with liver-dominant disease of varying primary tumor origins who were treated consecutively with ^90^Y resin microspheres (SIR-Spheres®, Sirtex Medical Limited, North Sydney, Australia) between January 2013 and November 2014. Most candidates for SIRT had either unresectable hepatocellular carcinoma (24.4 %) or unresectable liver-predominant metastases from colorectal cancer (25.6 %) and neuroendocrine tumors (21.8 %) or breast cancer (14.1 %) (Table 1). The decision to perform SIRT was guided in all cases by the well-defined published criteria for SIRT [18] as well as interdisciplinary consent after discussion with the liver tumor board. All patients provided written informed consent. This study was approved by our local ethics committee, University of Bonn-faculty of Medicine.

**Table 1:** Baseline patient and disease characteristics and treatment approach with SIRT according to the number of procedures with each application agent

	Sterile water	Isotonic 5 % glucose (G5W)	*p* value*
Gender, *n* (%)			ns
Male:female (procedure based)	28:22	33:21	
Male:female (patient based)	12:12	18:11	
Mean age, years			ns
Procedure based	61	65	
Patient based	60.5	66	
Older than 65 years old, *n* (%)			ns
Procedure based	16 (32 %)	25 (46.8 %)	
Patient based	7 (29 %)	15 (52 %)	
Primary tumor origin, *n* (%)			
Procedure based			
Colorectal cancer	15	14	ns
Hepatocellular carcinoma	11	14	
Neuroendocrine tumor	9	13	
Breast cancer	9	7	
Cholangiocarcinoma	4	2	
Others	2	4	
Patient based			
Colorectal cancer	5	7	
Hepatocellular carcinoma	6	7	
Neuroendocrine tumor	4	8	
Breast cancer	4	2	
Cholangiocarcinoma	3	1	
Others	2	4	
Prior TACE	6	9	ns
Activity planned GBq, median (range)			ns
Procedure based	1.54 GBq (0.5–2.5)	1.45 GBq (0.5–2.4)	
Patient based	1.5 (0.56–2.1)	1.4 (0.6–2.4)	
Activity delivered GBq, median (range)			
Procedure based	1.05 (0.2–2.29)	1.2 (0.3–2.4)	0.04
Patient based	1.0 (0.4–2.1)	1.4 (0.6–2.4)	0.005
Treatment approach, *n* (%)			ns
Whole-liver treatment	11	8	
Lobar	38	43	
Segmental	1	3	

*Chi-square test

### Pretreatment angiography and SIRT

The technique and rationale for the various procedures involved with delivering radioactive microspheres into the hepatic artery are well described elsewhere [19–22]. Apart from the change in application agent from sterile water to G5W for ^90^Y resin microspheres, the process for the pretreatment review, angiography, dosimetry and treatment procedure for SIRT, and the clinical team were otherwise identical.

All patients underwent preparatory hepatic arteriography to define the vascular anatomy of the liver and to assess the vascularity of hepatic tumors 1–2 weeks prior to SIRT. This was combined with technetium-99m-labelled human serum albumin (^99m^Tc-HSA) simulation and single-photon emission CT (SPECT) imaging to rule out any shunting to the lung and to detect non-target delivery to the gastrointestinal tract, which was corrected, prior to delivery of SIRT. The prescribed activity was calculated principally using the body surface area method based on the target volumes of the tumor and liver for each patient [18].

On the treatment day, ^90^Y resin microspheres were administered after confirming that there were no new collateral vessels connecting to the gastrointestinal tract. SIRT was performed under constant fluoroscopic guidance; eminent stasis or backflow led to the termination of treatment irrespective of the amount of activity given at that time point. Either sterile water or G5W was used to prime the delivery set then suspend the ^90^Y resin microspheres. An aliquot of the ^90^Y resin microsphere suspension was loaded into the A-line for deposition into the hepatic artery and flushed through with 2 mL sterile water (or G5W) followed by 2 mL non-ionic CM and finally 3–5 mL sterile water (or G5W). These steps were repeated until the prescribed activity was delivered to the patient or stasis. Any residual activity was calculated according to a previously described method [23]. A residual activity less than or equal to 5 % was considered as 100 % delivery of the calculated activity. Within 24 h of SIRT, planar Bremsstrahlung scintigraphy (BS) of the upper abdomen followed by a SPECT/CT scan was performed in all patients [18].

#### Definition of early stasis and flow reduction

As mentioned above, an early stasis is defined as a lack of an antegrade flow at angiography during infusion. A significant flow reduction is defined as a reduction in flow, verified by interventional radiologists as the contrast column clear within two to five heartbeats [24].

### Statistical analysis

Variables of interest were calculated using descriptive statistics. The chi-square test (*χ*^2^) was used to compare different variables in the sterile water and glucose 5 % water (G5W) groups. For the comparison of variables with a normal distribution, a *t* test was used. Statistical analysis was performed using a commercially available software package (SPSS 20, IMB, Armonk, NY, USA). Values of *p* < 0.05 were considered significant. The data were analyzed not only according to the number of procedures but also as patient based.

## Results

### Patients and procedure

One hundred and four SIRT procedures were performed on 78 patients (45 male, mean age: 63 years old, range: 31–87 years old) with either unresectable HCC or cholangiocarcinoma or chemorefractory liver-dominant metastatic cancer (Table 1). Twenty-five patients underwent sequential therapy as a lobar treatment (32 %), some (*n* = 8) receiving their second procedure with G5W (Table 1). Fifty-three patients underwent only one procedure as lobar (in 42 patients (79.2 %)), whole-liver (10 patients (18.9 %)), or segmental (1 patient (1.9 %)) SIRT (Table 1). Because of inhomogeneity of the data between the patients with sequential treatments (e.g., developing stasis in one treated lobe or using glucose and water for different lobes in the same patient), we excluded these patients from the patient-based analysis. Before December 2013, 50 procedures were performed with sterile water on 41 patients (mean age: 61 years), and subsequently, 54 procedures were performed with G5W on 37 patients (mean age: 65 years). There were no significant differences identified between the treatment groups for any baseline characteristics: either patient, tumor type (prior treatment—all patients with metastases had chemorefractory disease), or SIRT procedure. The mean lung shunt was 3.2 % (0.7–15 %).

### Flow reduction, stasis, and administered activity

Tables 2 and 3 summarize the results from our analyses. Stasis or significant flow reduction occurred in 56 and 20 % of procedures as well as 58.3 and 6.9 % of patients with sterile water and G5W, respectively (*p* < 0.0001). A stasis alone occurred in 28 and 11 % of procedures as well as 29.2 and 6.9 % of patients with sterile water and G5W, respectively (*p* = 0.02 and 0.03, respectively).

**Table 2:** Post-treatment parameters according to number of procedures with each application agent for SIRT

	Sterile water	Isotonic 5 % glucose (G5W)	*p* value*
	(*n* = 50)	(*n* = 54)	
Stasis or flow reduction, *n* (%)			<0.0001
No	22 (44 %)	43 (80 %)	
Yes	28 (56 %)	11 (20 %)	
Stasis, *n* (%)			0.02
No	36 (72 %)	48 (89 %)	
Yes	14 (28 %)	06 (11 %)	
Mean activity delivered (as a percentage of planned activity)	77.4 ± 24.3 %	96.1 ± 11.0 %	<0.001
Activity delivered (as a percentage of planned activity), *n* (%)			<0.0001
100 %	22 (44 %)	47 (87 %)	
90–95 %	04 (8 %)	01 (2 %)	
80–89 %	03 (6 %)	01 (2 %)	
50–79 %	15 (30 %)	5 (9 %)	
<50 %	6 (12 %)	0 (0 %)	
Mild-to-moderate abdominal pain during application of SIRT, *n* (%)			<0.0001
Yes	22 (44 %)	1 (1.8 %)	
No	28 (56 %)	53 (98.2 %)	

*Chi-square test

**Table 3:** Post-treatment parameters according to number of patients with each application agent for SIRT

	Sterile water	Isotonic 5 % glucose (G5W)	*p* value*
Stasis or flow reduction, *n* (%)			<0.0001
No	10 (41.7 %)	27 (93.1 %)	
Yes	14 (58.3 %)	02 (6.9 %)	
Stasis, *n* (%)			0.03
No	17 (70.8 %)	27 (93.1 %)	
Yes	7 (29.2 %)	02 (6.9 %)	
Mean activity delivered (as a percentage of planned activity)	74.7 ± 25.9 %	99.6 ± 1.9 %	<0.001
Activity delivered (as a percentage of planned activity), *n* (%)			<0.0001
100 %	8 (33.4 %)	28 (96.6 %)	
90–95 %	04 (16.7 %)	01 (3.4 %)	
80–89 %	02 (8.3 %)	0 (0 %)	
50–79 %	6 (25 %)	0 (0 %)	
<50 %	4 (16.7 %)	0 (0 %)	
Mild-to-moderate abdominal pain during application of SIRT, *n* (%)			0.002
Yes	9 (37.5 %)	1 (3.4 %)	
No	15 (62.5 %)	28 (96.6 %)	

*Chi-square test

The administration of the whole calculated activity with air shot at the end of the procedure was possible in 11 of 50 (22 %) and 46 of 54 (85 %) procedures as well as in 5 of 24 (20.8 %) and 28 of 29 (96.6 %) of patients with sterile water and G5W, respectively (*p* < 0.0001). According to all the procedures, because of flow reduction or stasis, a mean of 77.4 ± 24.3 % and 96.1 ± 11.0 % of the calculated activity was administered in the sterile water and G5W groups, respectively (*p* < 0.001). In the patient-based analysis, it was 74.7 ± 25.9 % and 99.6 ± 1.9 % in the sterile water and G5W groups, respectively (*p* < 0.001). A prior TACE did not have any effect on early flow reduction or stasis (*p* = 0.46).

### Safety

Over at least the 3-month follow-up post-procedure(s), no patient developed gastroduodenal ulcer or radioembolization-induced liver disease (REILD). Mild-to-moderate upper abdominal pain occurred in 22 of 50 (44 %) and 1 of 54 (1.8 %) procedures as well as 9 of 24 (37.5 %) and 1 of 29 (3.4 %) with sterile water and G5W, respectively (*p* < 0.0001 and 0.002, respectively).

## Discussion

This retrospective review of patients from the same center found that the incidence stasis during SIRT was significantly reduced when using non-ionic CM and isotonic glucose 5 % water (G5W) for the administration of ^90^Y resin microspheres rather than sterile water. With a lower incidence stasis, a higher percentage of the planned activity for ^90^Y was administered using G5W as the delivery medium (compared with sterile water). Furthermore, significantly fewer patients reported mild-to-moderate pain and discomfort during the procedure. This analysis provides preliminary data to support our hypothesis that the administration of ^90^Y resin microspheres with an isotonic solution decreases the likelihood of stasis (probably due to a reduction in vasospasm) during SIRT. This finding is congruent with the delivery of other intra-arterial therapies including glass microspheres (which uses isotonic saline solution). None of the patients in this case series experienced gastrointestinal events due to the non-target delivery of SIRT, and so, our contention that G5W may also reduce the non-target delivery of ^90^Y resin microspheres due to reflux needs to be evaluated further in a larger cohort of patients.

These are encouraging findings since stasis not only prohibits the forward flow (and delivery of the entire planned activity of ^90^Y) but may not improve, and so, treatment with SIRT may need to be halted before completion of the prescribed activity. This is of concern particularly when less than 50 % of the planned activity of ^90^Y is delivered (as in 12 vs. 0 % of the sterile water and G5W procedures, also in 16.7 and 0 % of the patients in this study, respectively). Fortunately, stasis resulting in a significant reduction in the administration of the planned activity is a relatively rare event. In a study of 680 patients who received SIRT with ^90^Y resin microspheres using sterile water, 1.1 ± 0.06 GBq (92 %) of the 1.2 ± 0.06 GBq of the mean (±SD) planned activity was administered [25]. In a further study of 606 patients (also using sterile water), a median 1.17 GBq (94 %) of 1.25 GBq of the planned activity was administered with the first SIRT procedure and a median of 0.66 GBq (92 %) of 0.72 GBq with the second SIRT procedure; notably, in this second published series, the delivered activity did not appear to be a function of the planned activity (i.e., higher planned activities did not markedly increase the risk of stasis or diminish the percentage activity delivered).

Given the small volume of microspheres contained in a vial, the majority of the microspheres are infused after the first few milliliters of sterile water or G5W solution. This is a relatively low treatment volume compared with previous chemotherapy or loco-regional treatments (such as TACE). In an in vivo pig model, clustering of ^90^Y resin microspheres was rarely observed and more likely to be caused by intra-procedural arterial spasm rather than the inherent tendency of the microspheres to aggregate [8]. This supposition is supported by in vivo studies showing the injurious effects of sterile water on the vascular endothelium leading to vasospasm [16, 17].

Unlike stasis, observed reductions in flow (characterized by diminished clearing of CM during SIRT) often improves. It is important to consider whether the incidence of stasis and reductions in the flow may also be a function of the injection speed and the skill and experience of the interventional radiologists (although, in this study, we tried to minimize this factor by using the same clinician and injection technique throughout the study). The use of microcatheters also permits the administration of ^90^Y resin microspheres at low and consistent flow rates while at the same time enabling adequate flow rates and particle suspension [10]. Other factors which may impact on the effective delivery of SIRT is the number of prior lines of chemotherapy—since patients who have recently received chemotherapy have vessels prone to dissection and spasm. In addition, diminished cardiac output in elderly patients may result in slower than expected hepatic arterial flow. Even though steps were taken to verify the similarity of each of our comparator groups in this analysis, further prospective evaluation in larger cohorts of patients is needed to ratify our findings.

## Conclusions

In conclusion, using non-ionic contrast and isotonic glucose 5 % (G5W) during the administration of ^90^Y resin microspheres rather than sterile water appears to favorably impact on the safety of SIRT, eliminate and/or minimize flow reductions/stasis during administration, and improve percentage activity delivered as well as the ease of delivery of ^90^Y resin microspheres and improved peri-procedural patient comfort.

## References

[1] Sarfaraz M, Kennedy AS, Lodge MA, Li XA, Wu X, Yu CX (2004). Radiation absorbed dose distribution in a patient treated with yttrium-90 microspheres for hepatocellular carcinoma. Med Phys.

[2] Kennedy AS, Nutting C, Coldwell D, Gaiser J, Drachenberg C (2004). Pathologic response and microdosimetry of (90)Y microspheres in man: review of four explanted whole livers. Int J Radiat Oncol Biol Phys.

[3] Campbell AM, Bailey IH, Burton MA (2001). Tumour dosimetry in human liver following hepatic yttrium-90 microsphere therapy. Phys Med Biol.

[4] Dezarn WA, Cessna JT, DeWerd LA, Feng W, Gates VL, Halama J (2011). Recommendations of the American Association of Physicists in Medicine on dosimetry, imaging, and quality assurance procedures for 90Y microsphere brachytherapy in the treatment of hepatic malignancies. Med Phys.

[5] Kennedy AS, Kleinstreuer C, Basciano CA, Dezarn WA (2010). Computer modeling of yttrium-90-microsphere transport in the hepatic arterial tree to improve clinical outcomes. Int J Radiat Oncol Biol Phys.

[6] Sangro B, Inarrairaegui M, Bilbao JI (2012). Radioembolization for hepatocellular carcinoma. J Hepatol.

[7] Wang LM, Jani AR, Hill EJ, Sharma RA (2013). Anatomical basis and histopathological changes resulting from selective internal radiotherapy for liver metastases. J Clin Pathol.

[8] Bilbao JI, de Martino A, de Luis E, Diaz-Dorronsoro L, Alonso-Burgos A, Martinez de la Cuesta A (2009). Biocompatibility, inflammatory response, and recannalization characteristics of nonradioactive resin microspheres: histological findings. Cardiovasc Intervent Radiol.

[9] MacKie S, de Silva S, Aslan P, Ladd L, Houang M, Cade D (2011). Super selective radio embolization of the porcine kidney with 90yttrium resin microspheres: a feasibility, safety and dose ranging study. J Urol.

[10] Kleinstreuer C, Basciano CA, Childress EM, Kennedy AS (2012). A new catheter for tumor targeting with radioactive microspheres in representative hepatic artery systems. Part I: impact of catheter presence on local blood flow and microsphere delivery. J Biomech Eng.

[11] Kennedy A, Nag S, Salem R, Murthy R, McEwan AJ, Nutting C (2007). Recommendations for radioembolization of hepatic malignancies using yttrium-90 microsphere brachytherapy: a consensus panel report from the radioembolization brachytherapy oncology consortium. Int J Radiat Oncol Biol Phys.

[12] Piana PM, Bar V, Doyle L, Anne R, Sato T, Eschelman DJ (2014). Early arterial stasis during resin-based yttrium-90 radioembolization: incidence and preliminary outcomes. HPB (Oxford).

[13] Nace GW, Steel JL, Amesur N, Zajko A, Nastasi BE, Joyce J (2011). Yttrium-90 radioembolization for colorectal cancer liver metastases: a single institution experience. Int J Surg Oncol.

[14] Krumbhaar EB (1914). Hemolysis due to intravenous injection of distilled water. JAMA.

[15] Tanimura A, Tanaka S, Kitazono M (1986). Superficial intimal injury of the rabbit carotid artery induced by distilled water. Virchows Arch B Cell Pathol Incl Mol Pathol.

[16] Collier J, Vallance P (1989). Endothelium-derived relaxing factor is an endogenous vasodilator in man. Br J Pharmacol.

[17] Sogo N, Wilkinson IB, MacCallum H, Khan SQ, Strachan FE, Newby DE (2000). A novel S-nitrosothiol (RIG200) causes prolonged relaxation in dorsal hand veins with damaged endothelium. Clin Pharmacol Ther.

[18] Ahmadzadehfar H, Biersack HJ, Ezziddin S (2010). Radioembolization of liver tumors with yttrium-90 microspheres. Semin Nucl Med.

[19] Sabet A, Ahmadzadehfar H, Muckle M, Haslerud T, Wilhelm K, Biersack HJ (2011). Significance of oral administration of sodium perchlorate in planning liver-directed radioembolization. J Nucl Med.

[20] Ahmadzadehfar H, Sabet A, Biermann K, Muckle M, Brockmann H, Kuhl C (2010). The significance of 99mTc-MAA SPECT/CT liver perfusion imaging in treatment planning for 90Y-microsphere selective internal radiation treatment. J Nucl Med.

[21] Ahmadzadehfar H, Mohlenbruch M, Sabet A, Meyer C, Muckle M, Haslerud T (2011). Is prophylactic embolization of the hepatic falciform artery needed before radioembolization in patients with (99 m)Tc-MAA accumulation in the anterior abdominal wall?. Eur J Nucl Med Mol Imaging.

[22] Ahmadzadehfar H, Muckle M, Sabet A, Wilhelm K, Kuhl C, Biermann K (2011). The significance of Bremsstrahlung SPECT/CT after yttrium-90 radioembolization treatment in the prediction of extrahepatic side effects. Eur J Nucl Med Mol Imaging.

[23] Ahmadzadehfar H, Haslerud T, Reichmann K, Meyer C, Habibi E, Fimmers R (2014). Residual activity after radioembolization of liver tumours with 90Y resin microspheres. A safe calculation method. Nuklearmedizin Nuclear medicine.

[24] Lencioni R, de Baere T, Burrel M, Caridi JG, Lammer J, Malagari K (2012). Transcatheter treatment of hepatocellular carcinoma with Doxorubicin-loaded DC Bead (DEBDOX): technical recommendations. Cardiovasc Intervent Radiol.

[25] Kennedy AS, McNeillie P, Dezarn WA, Nutting C, Sangro B, Wertman D (2009). Treatment parameters and outcome in 680 treatments of internal radiation with resin 90Y-microspheres for unresectable hepatic tumors. Int J Radiat Oncol Biol Phys.

